# Epigenetic and Immune-Cell Infiltration Changes in the Tumor Microenvironment in Hepatocellular Carcinoma

**DOI:** 10.3389/fimmu.2021.793343

**Published:** 2021-12-02

**Authors:** Zeng-Hong Wu, Dong-Liang Yang, Liang Wang, Jia Liu

**Affiliations:** ^1^ Department of Infectious Diseases, Union Hospital, Tongji Medical College, Huazhong University of Science and Technology, Wuhan, China; ^2^ Department of Urology, Union Hospital, Tongji Medical College, Huazhong University of Science and Technology, Wuhan, China

**Keywords:** hepatocellular carcinoma, epigenetic, inflammatory response, CRISPR, TCGA

## Abstract

**Background:**

Epigenetics regulate gene expression without altering the DNA sequence. Epigenetics targeted chemotherapeutic approach can be used to overcome treatment resistance and low response rate in HCC. However, a comprehensive review of genomic data was carried out to determine the role of epigenesis in the tumor microenvironment (TME), immune cell-infiltration characteristics in HCC is still insufficient.

**Methods:**

The association between epigenetic-related genes (ERGs), inflammatory response-related genes (IRRGs) and CRISPR genes was determined by merging genomic and CRISPR data. Further, characteristics of immune-cell infiltration in the tumor microenvironment was evaluated.

**Results:**

Nine differentially expressed genes (*ANP32B, ASF1A, BCORL1, BMI1, BUB1, CBX2, CBX3, CDK1*, and *CDK5*) were shown to be independent prognostic factors based on lasso regression in the TCGA-LIHC and ICGC databases. In addition, the results showed significant differences in expression of *PDCD-1* (*PD-1*) and *CTLA4* between the high- and low-epigenetic score groups. The CTRP and PRISM-derived drug response data yielded four CTRP-derived compounds (SB-743921, GSK461364, gemcitabine, and paclitaxel) and two PRISM-derived compounds (dolastatin-10 and LY2606368). Patients with high ERGs benefited more from immune checkpoint inhibitor (ICI) therapy than patients with low ERGs. In addition, the high ERGs subgroup had a higher T cell exclusion score, while the low ERGs subgroup had a higher T cell dysfunction. However, there was no difference in microsatellite instability (MSI) score among the two subgroups. Further, genome-wide CRISPR-based loss-of function screening derived from DepMap was conducted to determine key genes leading to HCC development and progression. In total, 640 genes were identified to be essential for survival in HCC cell lines. The protein-protein interaction (PPI) network demonstrated that IRRGs *PSEN1* was linked to most ERGs and CRISPR genes such as *CDK1, TOP2A, CBX2* and *CBX3.*

**Conclusion:**

Epigenetic alterations of cancer-related genes in the tumor microenvironment play a major role in carcinogenesis. This study showed that epigenetic-related novel biomarkers could be useful in predicting prognosis, clinical diagnosis, and management in HCC.

## Background

Hepatocellular carcinoma (HCC) is a highly aggressive malignant disease. It is the fastest-growing cause of cancer-related death worldwide (1). In addition, HCC is the most common form of primary liver cancer, accounting for 85-90% of all cases. The most common risk factors associated with HCC include hepatitis B virus (HBV), hepatitis C virus (HCV), aflatoxin exposure and nonalcoholic steatohepatitis. However, the molecular pathogenesis of HCC is still not clear (2, 3). Early-stage HCC presents with nonspecific manifestations. Therefore, diagnosing early-stage HCC is challenging. Approximately 60% of the patients experience recurrence or distant metastasis after surgery (4). Early-stage HCC is curable by resection, liver transplantation or ablation. However, most patients are diagnosed late with unresectable disease (5). Recent years have seen the advent of the role of immune checkpoint inhibitors has been investigated and the PD-L1 inhibitor atezolizumab in combination with bevacizumab has reported unprecedented results in HCC patients (6–8). Therefore, there is an urgent need to investigate the molecular pathogenesis and regulatory network of HCC to discover therapeutic targets and develop effective drugs. Further, there is a need to establish a model for predicting HCC prognosis.

Epigenetics describes heritable or inheritable mechanisms that regulate gene expression without altering the DNA sequence. Epigenetic modifications include DNA methylation, histone modification, chromatin remodeling, and modifications of RNA, including non-coding RNA (9). Aberrant epigenetic changes may alter the expression of oncogenes or tumor suppressor genes leading to tumorigenesis. A recent study showed that histone H3K27 demethylase *KDM6A* was an epigenetic gatekeeper for mTORC1 signaling in gastrointestinal cancers, such as liver and pancreatic cancers (10). Moreover, abnormal epigenetic changes may lead to phenotypic changes in tumor cell growth, immune escape, metastasis, heterogeneity and chemoresistance (11). In addition, some epigenetic changes may directly lead to tumor development. A previous study reported that epigenetic immunoediting could drive an acquired immune evasion program in most aggressive mesenchymal glioblastoma multiforme subtype through reshaping the tumor immune microenvironment (12). Therefore, epigenetic processes can be targeted as an auxiliary means of immunotherapy to overcome treatment resistance and the low response rate in HCC. A recent study showed that inflammation triggers epigenetic alterations in cancer cells and components of the tumor microenvironment (13). Chronic inflammation may lead to cirrhosis and thus lead to cancer development, progression, and metastasis. A previous study reported that chronic liver inflammation could lead to the development of hepatobiliary cancers (11). However, only a few studies have evaluated the role of epigenetic-related genes (ERGs) in HCC. Currently, CRISPR-cas9 screening is appearing as a powerful tool for precise medicine. Combining cas9 with pooled guide RNA libraries facilitates screening of genes that contribute to specific biologic phenotypes and diseases in a high-throughput way. In the present study a comprehensive review of genomic data was carried out to determine the role of epigenesis in the tumor microenvironment (TME), immune cell-infiltration characteristics and inflammatory response in HCC. Further, the study explored possible predictive genes combined with the cell viability by CRISPR-cas9 screening from the dependency map (DepMap) portal for HCC prognosis.

## Materials and Methods

### Data Collection

Data on RNA-sequencing, including 424 cases (50 normal tissues and 374 tumor samples, FPKM value), somatic mutation and copy number variation (CNV) was obtained from The Cancer Genome Atlas Liver Hepatocellular Carcinoma (TCGA-LIHC) database. In addition, RNA-sequence data were also obtained from GSE76427 (52 normal tissues and 115 tumor tissues). The FPKM values were converted to transcripts per kilobase million (TPM) values. Batch effect was corrected using “combat” algorithm based on SVA R package. This study combined RNA-sequencing data and clinical information from the GSE76427 and the International Cancer Genome Consortium (ICGC). We extracted 720 epigenetic-related genes from EpiFactors, a database for epigenetic factors, corresponding genes and products (14) **Table S1**. In total, 200 inflammatory response-related genes (IRRGs) were downloaded from the gene set enrichment analysis (GSEA) hallmark inflammatory response gene set **Table S2**. Project Achilles uses genome-scale CRISPR-Cas9 tool for gene knock out to identify candidate genes critical for cancer survival (15). Essential genes in HCC were identified using genome-wide CRISPR screening from the dependency map (DepMap) portal. Dependency scores for approximately 17,000 candidate genes were calculated using the CERES algorithm (15). Candidate genes were determined as crucial genes with a CERES score of <−1 across 75% of HCC cell lines. Further, the link between the identified candidate genes, the ERGs and the IRRGs was explored. Differential expression of genes (DEGs) was determined at FDR<0.01 and |log_2_FC|≥1.5 using R software, limma package. RNA-seq analyses based on the adequate tumor purity and the tumor purity estimates by single sample gene set enrichment analysis (ssGSEA) for tumor tissue samples from HCC.

### Unsupervised Clustering for ERGs

Unsupervised cluster analysis was performed based on DEGs to identify different epigenetic modification patterns and classify patients for further analysis. The consensus clustering algorithm determines the number and stability of the clusters. Consensus clustering was performed using the ConsenSuClusterPlus R package and repeated 1000 times to ensure the stability of the classification (16). Further, Gene Set Variation Analysis (GSVA) was performed using the “GSVA” R package to determine differences in biological processes between the epigenetic modification models. The gene set “c2.cp.kegg. V6.4. symbols” was obtained from MSigDB database for GSVA analysis. GSVA is a nonparametric, unsupervised method for estimating gene set enrichment variation from samples of expressed datasets.

### Generation of Epigenetic Score

The epigenetic score was used to evaluate individual epigenetic modifications with HCC outcomes. The epigenetic signature was constructed as follows: First, DEGs were standardized in all HCC samples, and the overlapping genes were identified. The patients were divided into several groups for subsequent analysis using unsupervised cluster analysis. The number and stability of the gene clusters was determined using consensus clustering algorithm. Second, univariate Cox regression analysis was used to determine the prognostic genes. Finally, principal component analysis (PCA) was used to determine the principal components. The epigenetic score was then determined using the formula (16): *epigenetic score* =Σ (*PC*1*i* + *PC*2*i*). Where i refers to the expression value of phenotype ERGs.

### Construction and Validation of the Prognostic ERGs Signature

Univariate and multivariate Cox regression analyses were used to evaluate the prognostic value of ERGs. Significant genes in the univariate and multivariate Cox regression analyses were selected as the characteristic genes. The risk score for individual patients was calculated as follows: e^sum (gene's expression×coefficient)^. The patients were then divided into high- or low-risk groups based on the median risk score cut-off value. Receiver operating characteristic (ROC) curves were used to predict the accuracy of the prognostic signatures. Kaplan–Meier survival curves were used to examine the effect of the signature on survival. The TCGA and GSE76427 databases were used in the training set, while ICGC data sets were used in the testing set. The prognostic signature for IRRGs was constructed similarly to the ERG signature.

### Drug Sensitivity of the High and Low-Risk Groups

Drug sensitivity of the high and low-risk groups was determined using the “pRRophetic” and “ISOpureR” packages (17). Expression profile data and somatic mutation data of human cancer cell lines (CCLs) were obtained from the Broad Institute-Cancer Cell Line Encyclopedia (CCLE) project based on the DepMap portal. Drug sensitivity data of the CCLs were obtained from the Cancer Therapeutics Response Portal (CTRP) and PRISM Repurposing dataset. The CTRP and PRISM datasets provide the area under the dose-response curve (area under the curve—AUC) values as a measure of drug sensitivity, with lower AUC values suggesting increased sensitivity. The detailed data processing flow and algorithm reference Chen et al.’s study (17). In addition, heatmaps were used to depict principal component differences of immune cells between the high- and low-risk groups based on XCELL, TIMER, QUANTISEQ, MCPCOUNTER, EPIC, CIBERSORT, and CIBERSORT-ABS algorithms.

### Statistical Analysis

All statistical analyses were performed in R statistical software version 3.6.2. Normally and not-normally distributed data were analyzed using the unpaired student's t-test and Wilcoxon rank-sum test, respectively. The relationship between the molecular signature and the clinicopathological parameters was assessed using the chi-square test. Logistic regression analyses were employed to determine whether the signature was an independent prognostic factor. The “surv-cutpoint” function was used to repeatedly test all possible cut points to find the maximum rank statistic, classified as the epigenetic score. The patients were divided into high and low-risk groups based on the maximum selected log-rank statistic to reduce the batch effect. The maftools package in R was used to analyze mutations in between the high- and low-risk groups. A *P-*value <0.05 was considered statistically significant.

## Results

### GSVA Analysis for ERGs

**Figure 1:**
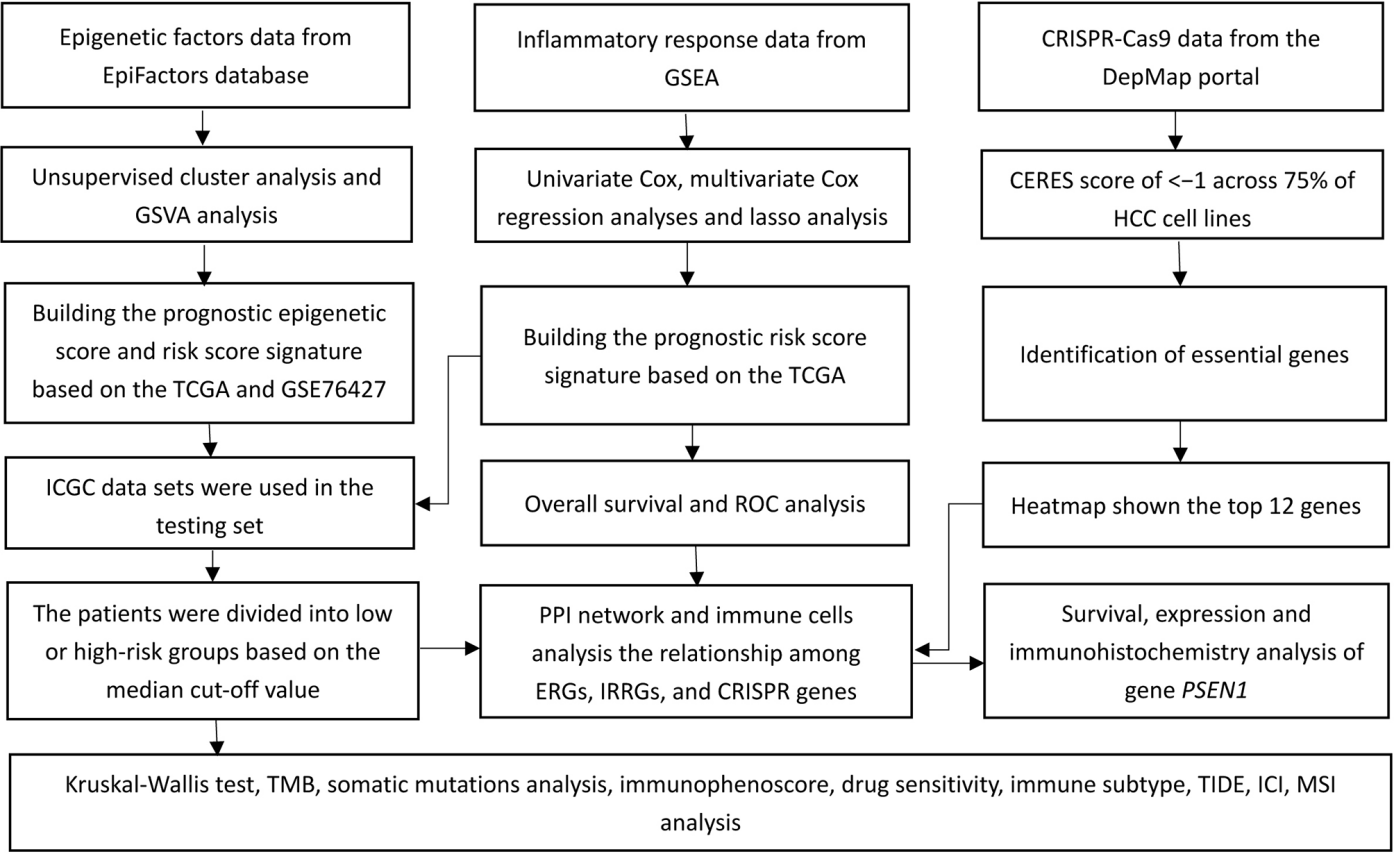
A flow chart of the study.

**Figure 2:**
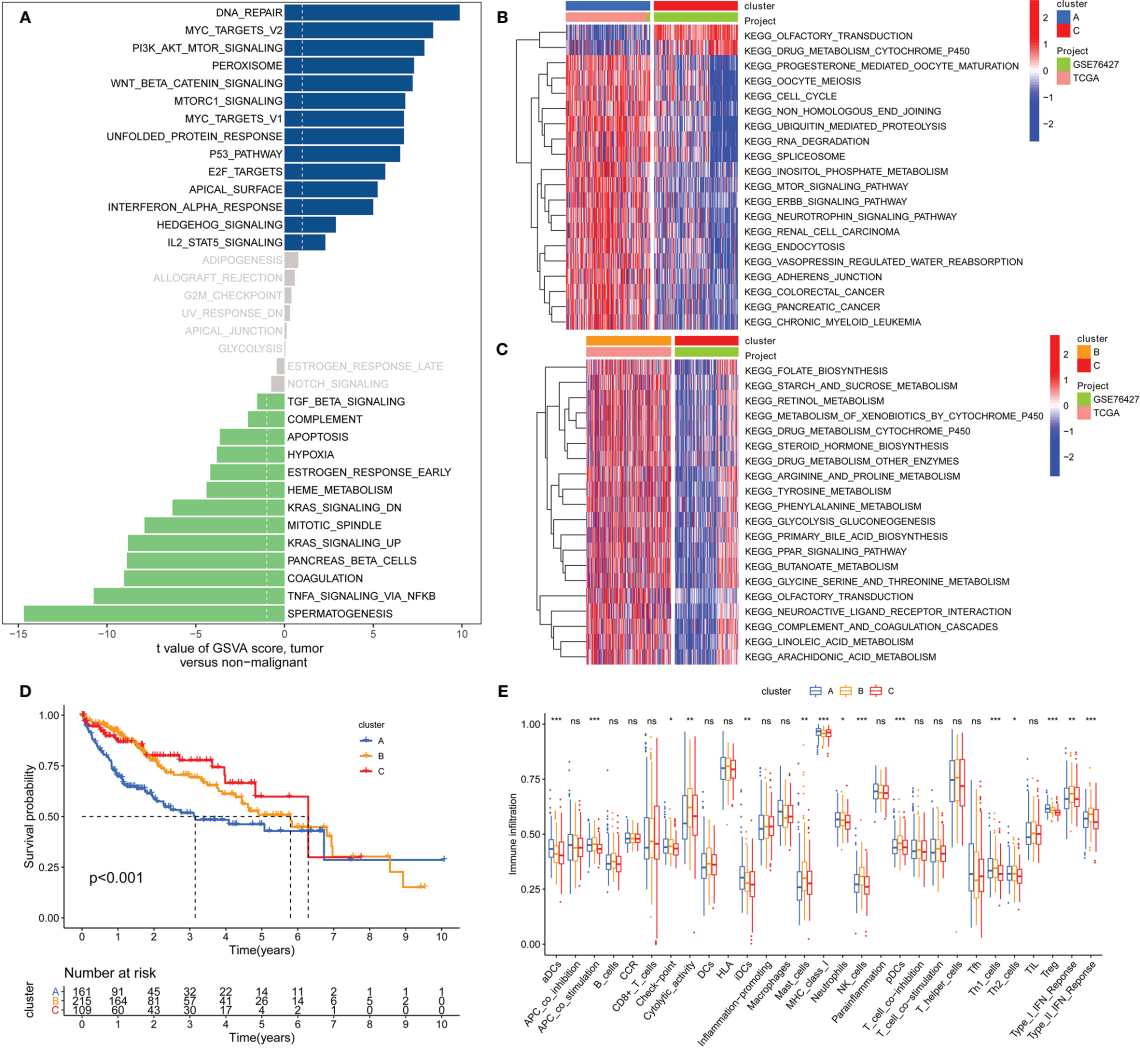
GSVA analysis and unsupervised clustering analysis based on differential expression of genes to identify different epigenetic modification patterns and classify patients for further analysis. **(A)** Enrichment of epigenetic-related genes (ERGs) in signaling pathways as determined by GSVA analysis; **(B)** Cluster-A was mainly enriched in pathways related to cancers such as mTOR signaling, ERBB signaling, cell cycle and some tumors; **(C)** Cluster-B was prominently linked to metabolism pathways such as linoleic acid metabolism, arachidonic acid metabolism and ether butanoate metabolism; **(D)** Kaplan–Meier analysis revealed that cluster-A model correlated with poor survival (Group A vs. Group B, *P*=0.001; Group B vs. Group C, *P*=0.491; Group A vs. Group C, *P*=0.002); **(E)** Determination of differences of immune cells among the three clusters based on the ssGSEA algorithm (The enrichment score is calculated by using the empirical cumulative distribution function). * indicate P < 0.05; ** indicate P < 0.01; *** indicate P < 0.001; ns indicate no significance.

**Figure 3:**
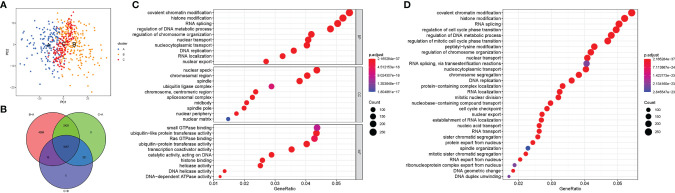
The differential expression of genes in each cluster and the function analysis of co-expressed genes among three clusters. **(A)** Principal component analysis for the transcriptome profiles of cluster A, B, and C; **(B)** Venn diagram showing the co-expressed genes in the three clusters; **(C)** GO analysis of the co-expressed genes in three clusters; **(D)** KEGG analysis of the co-expressed genes in three clusters and the result showed that the co-expressed genes were mainly enriched in pathways related to histone, cell cycle, DNA, and RNA functions.

**Figure 1** shows the study flow chart. The clinicopathological characteristics of the patients are presented in **Table 1**. The enriched ERGs signaling pathways were explored using GSVA analysis. The results showed that the ERGs were mainly enriched in cancer and immune-related pathways such as PI3K/AKT/mTOR, Wnt/beta-catenin, mTORC1, IL2/STAT5, TGF-beta, and TNF-alpha signaling pathways **Figure 2A**. Further, transcriptome data obtained from the TCGA and GEO databases were merged to obtain the DEGs of the 720 ERGs. Finally, using the unsupervised clustering method, three different clusters were identified, consisting of 162 cases of cluster-A, 215 cases of cluster-B, and 161 cases of cluster-C. GSVA enrichment analysis was then used to investigate the biological function of the different clusters. Cluster-A was mainly enriched in cancer-related pathways such as mTOR and ERBB signaling, and cell cycle progression **Figure 2B**. In contrast, Cluster-B was associated with metabolism pathways such as linoleic acid, arachidonic acid, and ether butanoate metabolism **Figure 2C**. Kaplan–Meier survival analysis showed that cluster-A was associated with poor survival **Figure 2D**. Therefore, we hypothesized that cluster A could affect epigenetics and other cancer-related processes, resulting in a poor prognosis. We then applied the ssGSEA to contrast immune cell differences between the three clusters **Figure 2E**. The results showed significant differences between cluster A and the other clusters in dendritic cells (DCs), neutrophils, MHC I, and regulatory T cells (Treg), suggesting that cluster A could play a key role in tumor immunity. Principal component analysis of the transcriptome profiles in the three clusters showed significant transcriptome differences **Figure 3A**. Further, the DEGs in each cluster were identified, and a Venn diagram was constructed to identify co-expressed genes. Finally, 5087 co-expressed genes in the three clusters were identified using the limma package **Figure 3B**. The KEGG analysis showed that the co-expressed genes were mainly enriched in pathways related to histone, cell cycle, DNA, and RNA functions **Figures 3C, D**. These findings indicate that epigenetic modifications play a key role in tumor development.

**Table 1:** The clinicopathological characteristics of HCC patients based on the TCGA, GEO and ICGC databases.

Clinical characteristics	TCGA (N = 377)	GSE76427 (N = 115)	ICGC (N = 260)
Age at diagnosis (y)	53 (16-90)	63.4 (14-93)	67.4 (31-89)
Futime (m)	28.0 (0-122.5)	21.9 (0.24-93.12)	26.5 (0.33-72)
Gender			
Female/Male	122/255	93/22	68/192
Stage			
I/II/III/IV/NA	175/87/86/5/24	55/34/22/3/1	40/117/80/23
Grade			
G1/G2/G3/G4/NA	55/180/124/13/5	NA	NA
T-classification			
T1/T2/T3/T4/TX/NA	185/95/81/13/1/2	NA	NA
M- classification			
M0/M1/MX	272/4/102	NA	NA
N- classification			
N0/N1/NX/NA	257/4/115/1	NA	NA
Status			
Alive/Death	129/248	92/23	214/192

Data express as Mean (min-max). NA, not applicable.

### The Epigenetic Score and Functional Annotation

**Figure 4:**
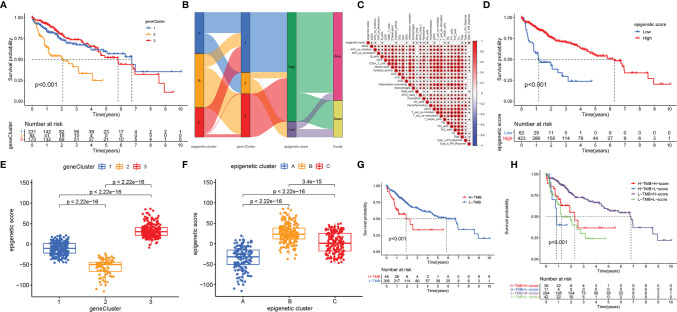
Construction of epigenetic scores; functional annotation and the relationship between the epigenetic score, epigenetic cluster and gene cluster. **(A)** Gene cluster 2 had a poor prognosis; **(B)** The ggalluvial diagram displaying relationships among epigenetic score, epigenetic cluster and gene cluster; **(C)** Correlations among infiltrating-cell types and epigenetic score; **(D)** The performance of epigenetic score in predicting patients’ prognosis. Patients with low epigenetic scores showed poor survival outcome; **(E)** Differences in epigenetic scores among epigenetic gene clusters as determined using the Kruskal-Wallis test; **(F)** Differences in epigenetic scores among the epigenetic clusters as revealed by Kruskal-Wallis test; **(G)** High TMB group had poor prognosis; **(H)** The survival results predicted by the combination of TMB and epigenetic score. * indicate P < 0.05.

Unsupervised cluster analysis based on the acquired 5087 epigenetic-related genes was used to classify patients into different groups. Three distinct clusters 1/2/3 were obtained. The analysis showed that the three gene clusters had different characteristic genes. Cluster 2 was associated with the worst prognosis **Figure 4A**. Considering the individual heterogeneity, an epigenetic score was established to quantify the epigenetic pattern in each patient based on the phenotypic related genes. A ggalluvial diagram was used to visualize the relationship between the epigenetic score, epigenetic cluster and gene cluster **Figure 4B**. The relationship between immune-cell infiltration and the epigenetic score is presented in **Figure 4C**. The patients were divided into low or high-risk groups based on the median cut-off value. A low epigenetic score was associated with poor survival **Figure 4D**. The Kruskal-Wallis test was used to determine significant differences between the epigenetic score, gene clusters, and epigenetic clusters. A low epigenetic score, gene cluster 2 and epigenetic cluster A were associated with poor survival **Figures 4E, F**. Previous studies have reported that tumor mutation burden (TMB) plays an important role in tumor prognosis. Thus, we explored the relationship between the high/low epigenetic score groups and TMB. The results showed that the high TMB group had a poorer prognosis. In addition, low/high TMB combined with a low epigenetic score was also associated with poor outcomes **Figures 4G, H**.

**Figure 5:**
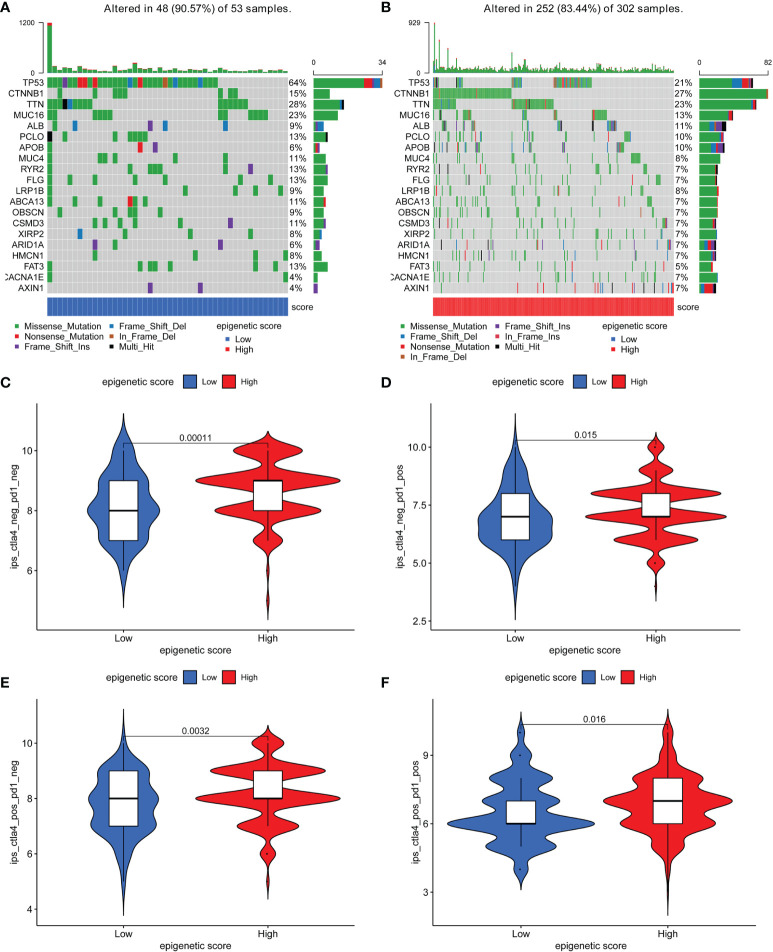
The distribution of somatic mutations among low and high epigenetic score groups and responsiveness to *CTLA-4* and *PD-1* therapy based on The Cancer Immunome Atlas (TCIA) database. **(A)** The distribution of somatic mutations among low epigenetic score group; **(B)** The distribution of somatic mutations among high epigenetic score group; **(C)** Negative PD-1 and Negative CTLA-4; **(D)** Positive PD-1 and Negative CTLA-4; **(E)** Negative PD-1 and Positive CTLA-4; **(F)** Positive PD-1 and Positive CTLA-4.

Further, the maftools package was used to analyze differences in somatic mutations between the low and high epigenetic score groups. The results indicated that the low epigenetic score group had increased TMB than the high epigenetic score group. *TP53* was the most common mutated gene **Figures 5A, B**. In addition, the relationship between the clinical-pathological characteristics and the epigenetic score was also explored. The results showed that a low score was associated with advanced tumor **Figure S1**. Finally, the immunophenoscore (IPS) based on The Cancer Immunome Atlas (TCIA) database was used to predict responsiveness to *CTLA-4* and *PD-1*. The results showed that the high- and low-score groups had a good response to *PD-1* and *CTLA-4* **Figures 5C–F**, which could provide useful insights for further exploration.

### Construction and Validation of the Prognostic ERGs Signature

Univariate Cox analysis identified 57 genes that were significantly associated with survival. A correlation coefficient for each gene in the model was calculated to determine possible collinearity between the genes **Table S3**. As a result, nine differentially expressed genes (*ANP32B, ASF1A, BCORL1, BMI1, BUB1, CBX2, CBX3, CDK1*, and *CDK5*) were selected as independent prognostic factors based on lasso regression in the TCGA-LIHC and ICGC databases **Table S4**. Moreover, the risk score for each patient was calculated, and the cohort was divided into two groups (high and low risk) based on the median cut-off value. Kaplan-Meier survival curves showed that the high-risk group had poorer survival than the low-risk group **Figure S2A**. In addition, ROC curves were used to determine whether the expression pattern could be used as an early predictor of HCC. The ROC curves showed AUC of 0.788, 0.699 and 0.722 for one, three- and five-years survival, respectively **Figure S2B**. Further, we established a risk survival status chart of the patients. The chart showed that the number of patients dying increased as the risk score increased **Figure S2C**. Moreover, the risk score was significantly associated with the tumor stage and T stage **Figure S2D**.

**Figure 6:**
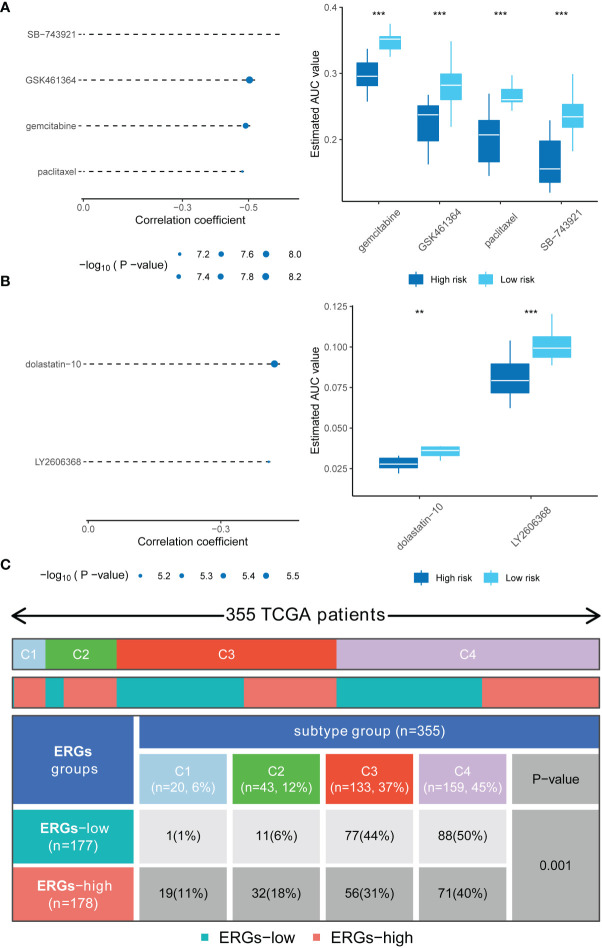
Drug sensitivity in high-risk score patients and the immune and molecular subtypes. **(A)** Four CTRP-derived compounds (including SB-743921, GSK461364, gemcitabine, and paclitaxel); **(B)** Two PRISM-derived compounds (including dolastatin-10 and LY2606368); **(C)** Relationship between immune subtypes and the risk signature. ** indicate P < 0.01; *** indicate P < 0.001.

Analyses of the CTRP and PRISM-derived drug response data yielded four CTRP-derived compounds (SB-743921, GSK461364, gemcitabine, and paclitaxel) and two PRISM-derived compounds (dolastatin-10 and LY2606368). The high-risk score group had lower estimated AUC values **Figures 6A, B**. The relationship between the nine genes and drug sensitivity was analyzed using the cellminer database [a relational database and query tool for the NCI-60 cancer cell lines (18)]. The results suggested that *CBX2* has the connection with most drugs such as dasatinib, acrichine, and nelarabine **Figure S3**.

**Figure 7:**
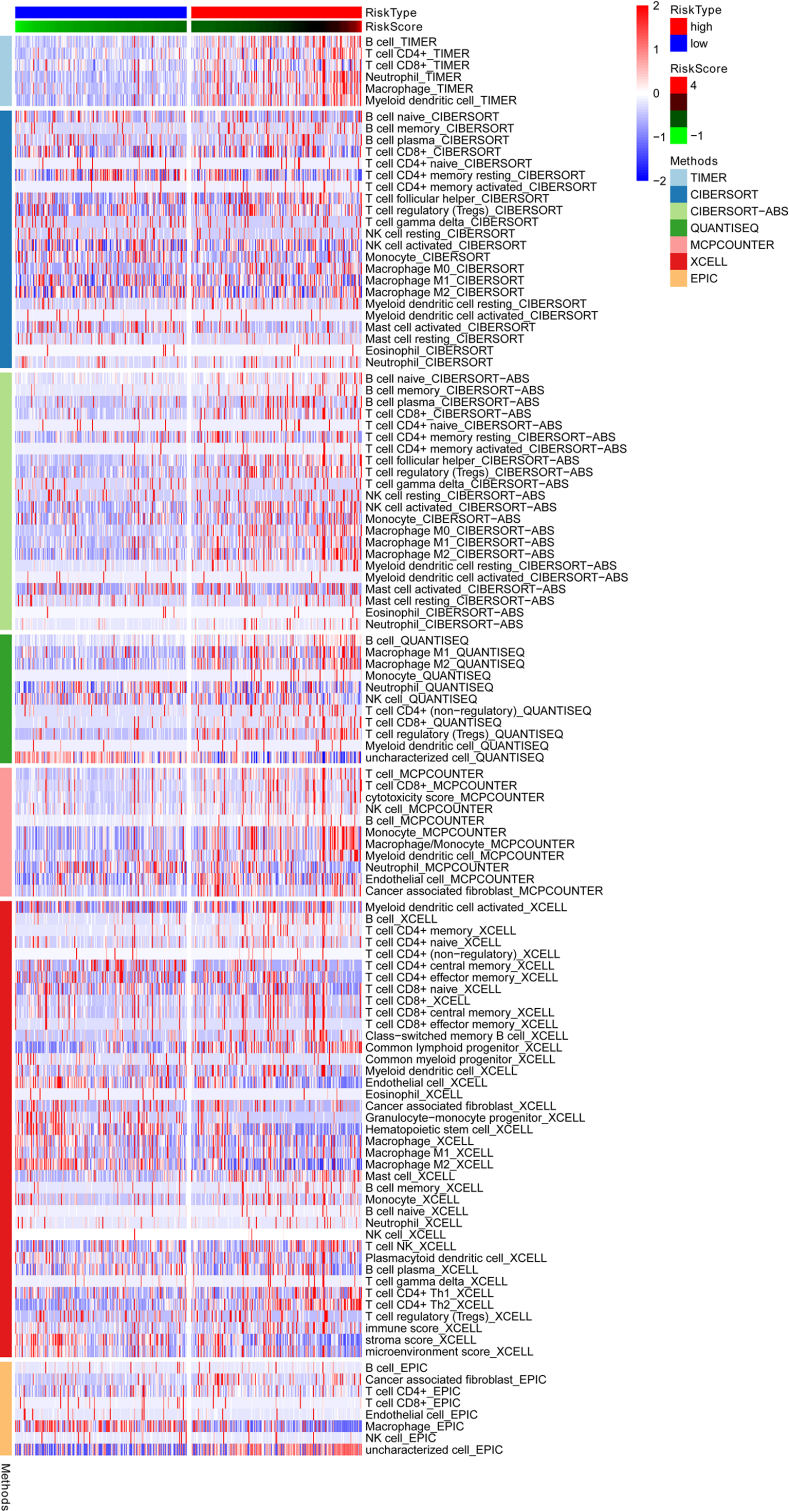
A heatmap showing the immune responses based on different algorithms in the low and high-risk groups and the results demonstrated that most immune cells showed a trend of high expression in the high-risk group when compared to low-risk group. TIMER, tumor immune estimation resource.

The relationship between ERGs groups and other immune and molecular subtypes were also explored. A total of 355 immune samples were further classified according to a pan-patient immune subtype (19) **Table S5**. As shown in **Figure 6C**, there were more C3 and C4 subtypes in the low ERGs subgroup. However, there were more C4 subtypes in the high ERGs subgroup (*P*=0.001, chi-square test). Further, Tumor Immune Dysfunction and Exclusion (TIDE) was used to evaluate the clinical efficacy of immunotherapy in different ERGs subgroups. A higher TIDE prediction score represents a higher likelihood of immune evasion, indicating that patients are less likely to benefit from immune checkpoint (ICI) therapy (20). In this study, patients with high ERGs benefited more from ICI treatment than patients with low ERGs **Figure S4A**. In addition, we found that the high ERGs subgroup had a higher T cell exclusion score. However, the low ERGs subgroup had a higher T cell dysfunction, with no differences in microsatellite instability (MSI) score between the two subgroups **Figures S4B-D**. Meanwhile, the ROC analysis suggested that the predictive value of the risk model was better than that of the 18-gene T-cell-inflamed signature (TIS) and TIDE models (21, 22) **Figure S4E**. Further, we explored differences in the expression of ICs between the two groups. The results showed significant differences in expression of *PDCD-1* (*PD-1*) and *CTLA4* between the two groups **Figure S5A**. In addition, there were significant differences in the expression of *RBM15, YTHDC1*, and *TDHDC2* between the high and low-risk groups **Figure S5B**. The heatmap showing immune responses based on different algorithms is shown in **Figure 7**. The results demonstrated that most immune cells showed a trend of high expression in the high-risk group when compared to low-risk group.

### Construction of the Prognostic IRRGs Signature

Univariate Cox analysis identified 50 genes that were significantly associated with survival **Table S6**. Further, multivariate Cox regression analyses identified 18 independent prognostic genes based on lasso regression **Table S7**. After that, the IRRGs signature was constructed. Kaplan-Meier survival curves showed that the high-risk group had poorer survival and an AUC value of 0.817, 0.769, and 0.754 for one, three, and five years survival, respectively. Univariate and multivariate Cox analysis revealed that the signature (HR: 3.797, 95CI: 2.744-5.255) and tumor stage (HR: 1.399, 95CI: 1.125-1.739) were independent prognosis factors of OS in HCC patients **Figure S6**. The results of the training set in the present study were consistent with results of the testing set in ICGC **Figure S7**. These results suggest that the IRRGs signature was stable and accurate and could be applied in the clinical management of HCC patients.

### Identification of Essential Genes by CRISPR 

**Figure 8:**
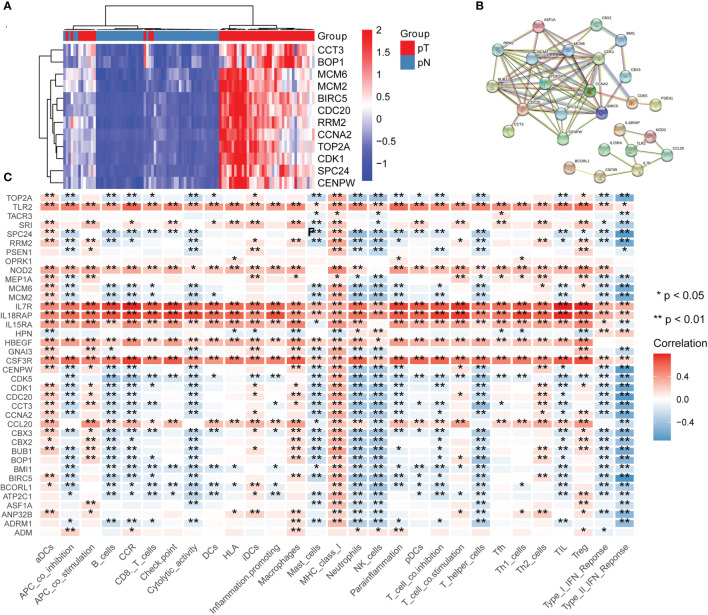
The relationship between epigenetic-related genes, inflammatory response-related genes and CRISPR genes. **(A)** A heatmap showing the top 12 genes in CRISPR, pT: tumor, pN: normal; **(B)** A protein-protein interaction (PPI) network demonstrated that IRRGs *PSEN1* was linked to most ERGs and CRISPR genes such as *CDK1, TOP2A, CBX2, and CBX3*; **(C)** The relationship between immune cells and these genes.

**Figure 9:**
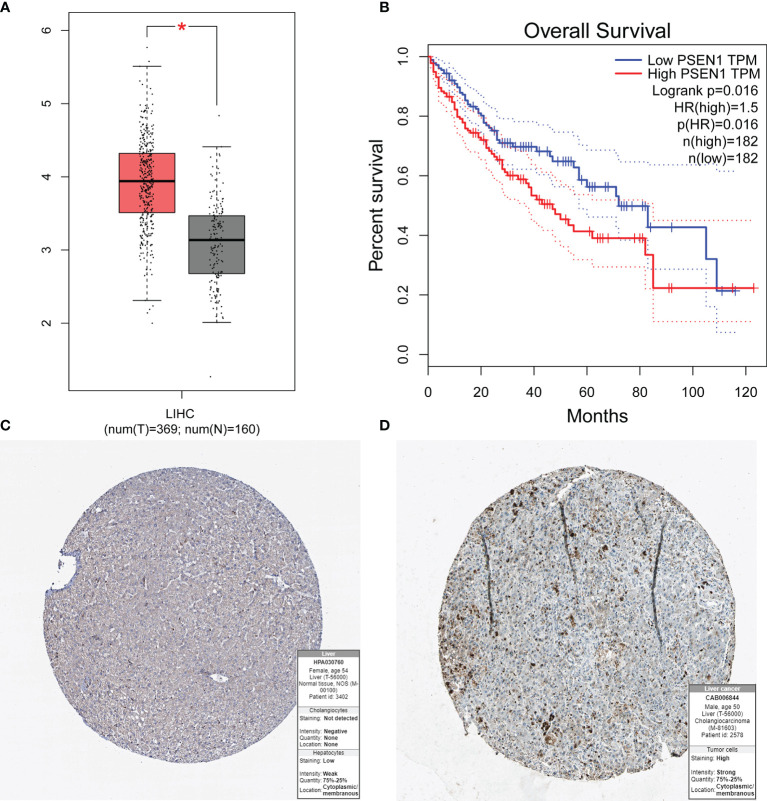
The expression of *PSEN1* in HCC and the prognosis based on the GEPIA and HPA databases. **(A)** High expression of PSEN1 based on the GEPIA database; **(B)** Overall survival analysis based on the GEPIA database; **(C)** The normal liver tissues based on the HPA database; **(D)** The tumor liver tissues based on the HPA database. * indicate P < 0.05.

To determine key genes responsible for HCC malignancy, we explored genome-wide CRISPR-based loss-of function screens derived from DepMap. In total, 640 genes were identified as essential in the survival of HCC cell lines **Table S8**. The heatmap showing the top 12 genes (*CDC20, TOP2A, BIRC5, RRM2, CCNA2, MCM2, BOP1, SPC24, CCT3, MCM6, CENPW*, and *CDK1*) is shown in **Figure 8A**. Finally, we evaluated the relationship between the ERGs, IRRGs, and CRISPR genes. The protein-protein interaction (PPI) network demonstrated that IRRGs *PSEN1* was linked to most ERGs and CRISPR genes such as *CDK1, TOP2A, CBX2*, and *CBX3* **Figure 8B**. Most genes were also shown to have a strong correlation with the immune cells **Figure 8C**. In addition, *PSEN1* was over expression in HCC and high expression of *PSEN1* indicated bad prognosis based on the GEPIA database **Figures 9A, B**. Immunohistochemistry results from the HPA database to illustrate that *PSEN1* were significantly increased in tumor tissue **Figures 9C, D**.

## Discussion

A combination of immunotherapy, radiotherapy, chemotherapy, and targeted therapy can be employed to suppress tumor progression and improve prognosis in HCC. In the next few years, cancer immunotherapy based on high-throughput sequencing data can accelerate the realization of precise cancer treatment. The development of tumors is complex. Epigenetics changes, immune-cell infiltration and the inflammatory response are among the factors involved. Epigenetic modification of the histone protein controlling differentiation and functions in Tregs play an important role in the TME (23). In this study, we first explored epigenetic changes in cancer-related genes and immune cell infiltration in HCC. The study may assist in our understanding of the epigenetic status and antitumor immune response in HCC and thus provide potential biomarkers for clinical therapeutic intervention.

Nine differentially expressed genes (*ANP32B, ASF1A, BCORL1, BMI1, BUB1, CBX2, CBX3, CDK1*, and *CDK5*) were shown to be independent prognostic factors in HCC. The acidic (leucine-rich) nuclear phosphoprotein 32 family member B (*ANP32B*), belong to the acidic nuclear phosphoprotein 32 (ANP32) family. *ANP32B* can modulate phosphorylation of *Bad* and expression of *Bak/Bax*, thus regulating apoptosis in HCC (24). Repair of damaged DNA relies on nucleosome dismantling of the nucleosome by histone chaperones and de-phosphorylation events carried out by Protein Phospatase 2A (PP2A) (25). Anti-silencing function 1a (*ASF1a*) is a histone H3-H4 chaperone isoform that contribute to chromatin assembling and transcription regulation. It is necessary in telomerase reverse transcriptase (TERT) expression, a factor required for immortalization of tumor cells (26). The *BCL6* corepressor-like 1 (*BCORL1*) is a transcriptional corepressor, which promotes cell migration and invasion by E-cadherin repression-induced epithelial-mesenchymal transition in HCC (27). *BMI1* is highly expressed in one-third of HCC patients and acts as an oncogene in hepatocarcinogenesis (28). A previous study also reported that long noncoding RNA lncAY could promote *BMI1* expression triggering signaling through the Wnt/β-catenin pathway in HCC (29). Another study found that lncRNA DUXAP8 could serve as a sponge of miR-490-5p enhancing expression of benzimidazoles 1 (*BUB1*) in HCC (30). Stable depletion of *BUB1* in ∼95% of human cells could influence entire chromosome segregation fidelity (31).

Chromobox 2 (*CBX2*), a chromobox family protein, is an essential component of the polycomb group complex. Knockdown of *CBX2* in HCC was shown to increase cell apoptosis and inhibit expression of WTIP, an inhibitor of the Hippo pathway (32). Chromobox protein homolog 3 (*CBX3*) is highly expressed in HCC tissues and is related to malignancy clinicopathological characteristics (33). The *CBX3* was shown to promote gastric cancer progression and was associated with chemotherapy and immunotherapy response (34). The cyclin-dependent kinase 1 (*CDK1*) drives cell division and inhibition of *CDK1* altered histone-modification of embryonic stem cells (35). Cyclin-dependent kinase-5 (CDK5) is a proline-directed serine/threonine kinase that play a key role in cancer progression (36). The ERGs identified in this study could play a key role in carcinogenesis. Further, this study provides useful insights for future exploration.

Inflammation plays a key role in cancers. Inflammatory response refers to the inflammatory microenvironment, which subverts the anti-tumor immune response by promoting angiogenesis and metastasis, and reduces sensitivity of tumor cells to chemotherapeutic agents. Epigenetics may be involved in the occurrence of inflammation by regulating inflammation-related genes. In this study, 18 IRRGs were identified and a prognostic signature was constructed. Further, 12 key genes responsible in the development and progression of HCC were identified based on CRISPR and *CDC20* with the largest log_2_FC. A recent study found that *CDC20* was over expressed in HCC contributing to radio resistance in cells with P53 mutation through the Bcl-2/Bax signaling pathway (37). *CDC20* is essential for H3K9 methylation and heterochromatin function (38). In addition, *PSEN1* was identified as the hub gene among the IRRGs, ERGs and CRISPR. Presenilin 1 (*PSEN1*) is the catalytic core of the γ-secretase complex that conducts the intramembranous proteolytic excision of multiple transmembrane proteins (39). *PSEN1* plays an important role in tumor radio resistance, induce cell cycle arrest, and stimulate DNA damage response (40). Moreover, most immune cells were shown to be highly correlated to the identified key genes (41). In this study, several gene biomarkers were explored to determine their effect on patient outcomes. However, further independent studies are required to confirm these results. This study had some limitations. First, the results were not validated in clinical samples. Second, the study had a small sample size which could affect reliability of the findings. Further studies are required to develop a prognostic model in HCC.

## Conclusion

Epigenetic changes of cancer-related genes in the tumor microenvironment could play significant roles in carcinogenesis. This study shows that epigenetic novel biomarkers could be useful in predicting prognosis, clinical diagnosis, and management in HCC.

## Data Availability Statement

Publicly available datasets were used in this study. This data can be found here: https://www.ncbi.nlm.nih.gov/geo/query/acc.cgi?acc=GSE76427.

## Author Contributions

Z-HW designed and analyzed the research study. Z-HW, D-LY, LW, and JL wrote and revised the manuscript, and collected the data. All authors contributed to the article and approved the submitted version.

## Funding

This work was supported by the National Natural Science Foundation of China (81861138044, 82172256, 81461130019, 8181101231, 91642118, 91742114), National Science and Technology Major Project (2017ZX10202202-001-009, 2017ZX10202202-002-008, 2017ZX10202201-002-003, 2017ZX10202203-007-006), Deutsche Forschungsgemeinschaft (Transregio TRR60), Integrated Innovative Team Project for Major Human Diseases Program of Tongji Medical College, Huazhong University of Science and Technology (HUST), The Double First-Class Disciplines Program of HUST (International Joint Laboratory for Infection and Immunity), International Cooperation Base of Hubei Province for Infection and Immunity, the Tongji-Rongcheng Center for Biomedicine, Huazhong University of Science and Technology, and the Medical Faculty of the University of Duisburg-Essen and Stiftung Universiätsmedizin, University Hospital Essen, Germany.

## Conflict of Interest

The authors declare that the research was conducted in the absence of any commercial or financial relationships that could be construed as a potential conflict of interest.

## Publisher’s Note

All claims expressed in this article are solely those of the authors and do not necessarily represent those of their affiliated organizations, or those of the publisher, the editors and the reviewers. Any product that may be evaluated in this article, or claim that may be made by its manufacturer, is not guaranteed or endorsed by the publisher.

## Supplementary Material

Supplementary Table 1 The 720 epigenetic-related genes obtained from the EpiFactors database.

Supplementary Table 2 The 200 inflammatory response-related genes based on gene set enrichment analysis (GSEA) online database.

Supplementary Table 3 Univariate Cox analysis identified 57 epigenetic-related genes.

Supplementary Table 4 Multivariate Cox regression analyses identified 9 differentially expressed genes which were found to be an independent prognostic factor based on lasso regression.

Supplementary Table 5 The 355 immune samples classified according to pan-patient immune subtypes.

Supplementary Table 6 Univariate Cox analysis identified 50 inflammatory response-related genes (IRRGs).

Supplementary Table 7 Multivariate Cox regression analyses identified 18 IRRGs as an independent prognostic gene based on lasso regression analysis.

Supplementary Table 8 A total of 640 genes were identified to play an important role in maintaining survival of HCC cell lines using CRISPR.
