# Supplementary material for: Epigenetic and Immune-Cell Infiltration Changes in the Tumor Microenvironment in Hepatocellular Carcinoma
Source: Front Immunol. 2021 Dec 2;12:793343. doi: 10.3389/fimmu.2021.793343 (PMC8674919; doi:10.3389/fimmu.2021.793343)

**Epigenetic factors signature and tumor microenvironment infiltration characterization in hepatocellular carcinoma**

**Zeng-Hong Wu^1^, MD; Dong-Liang Yang^1^, MD, Ph.D.; Jia Liu, MD^1^.**

^1^Department of Infectious Diseases, Union Hospital, Tongji Medical College, Huazhong University of Science and Technology, Wuhan 430022, China.

**Correspondence:**

**Jia Liu,** Department of Infectious Diseases, Union Hospital, Tongji Medical College, Huazhong University of Science and Technology, Wuhan 430022, China. (Email: jialiu77@hust.edu.cn)

**Figure S1.** The relationship between the clinical characteristics and epigenetic score.


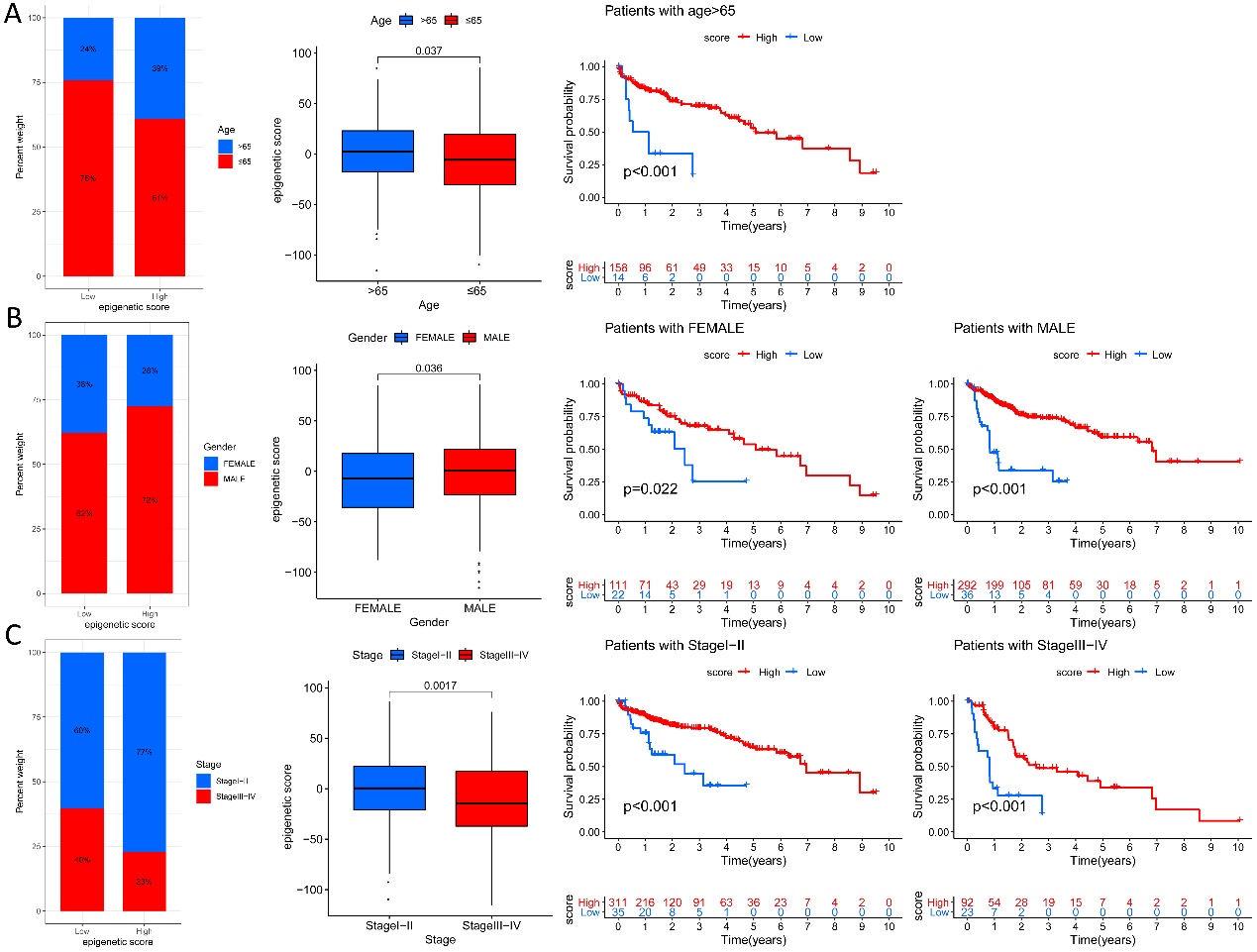


**Figure S2.** The characteristic of the epigenetic-related genes signature. A. Kaplan-Meier curves indicated that high-risk patients had poorer survival than the low-risk group; B. ROC curves to determine whether the expression pattern can be used as an early predictor of HCC; C. The risk survival status chart of patients; D. The connection between clinical pathology and risk scores.


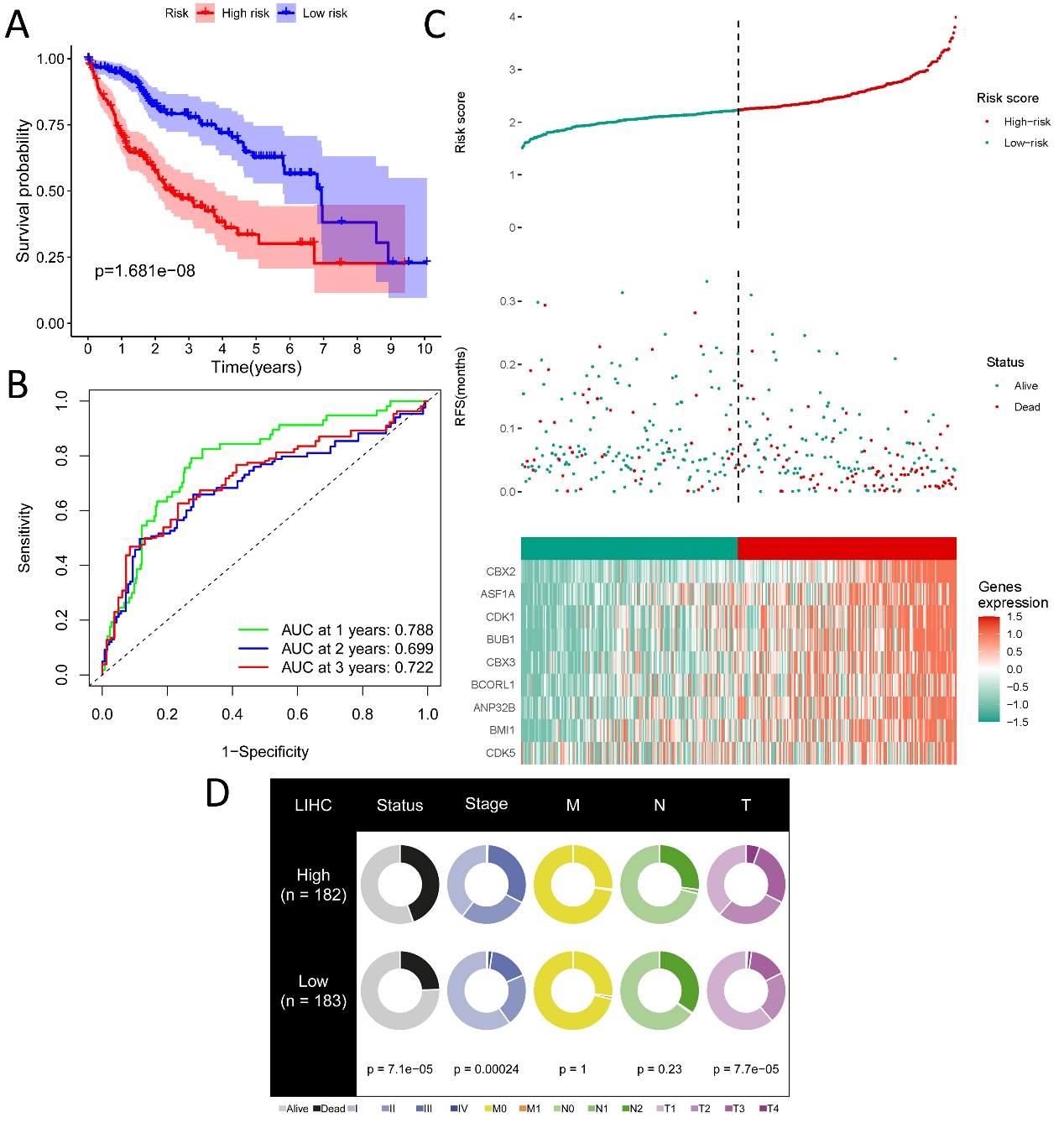


**Figure S3.** The relationship between 9 epigenetic-related genes and the drug sensitivity based on the cellminer data.


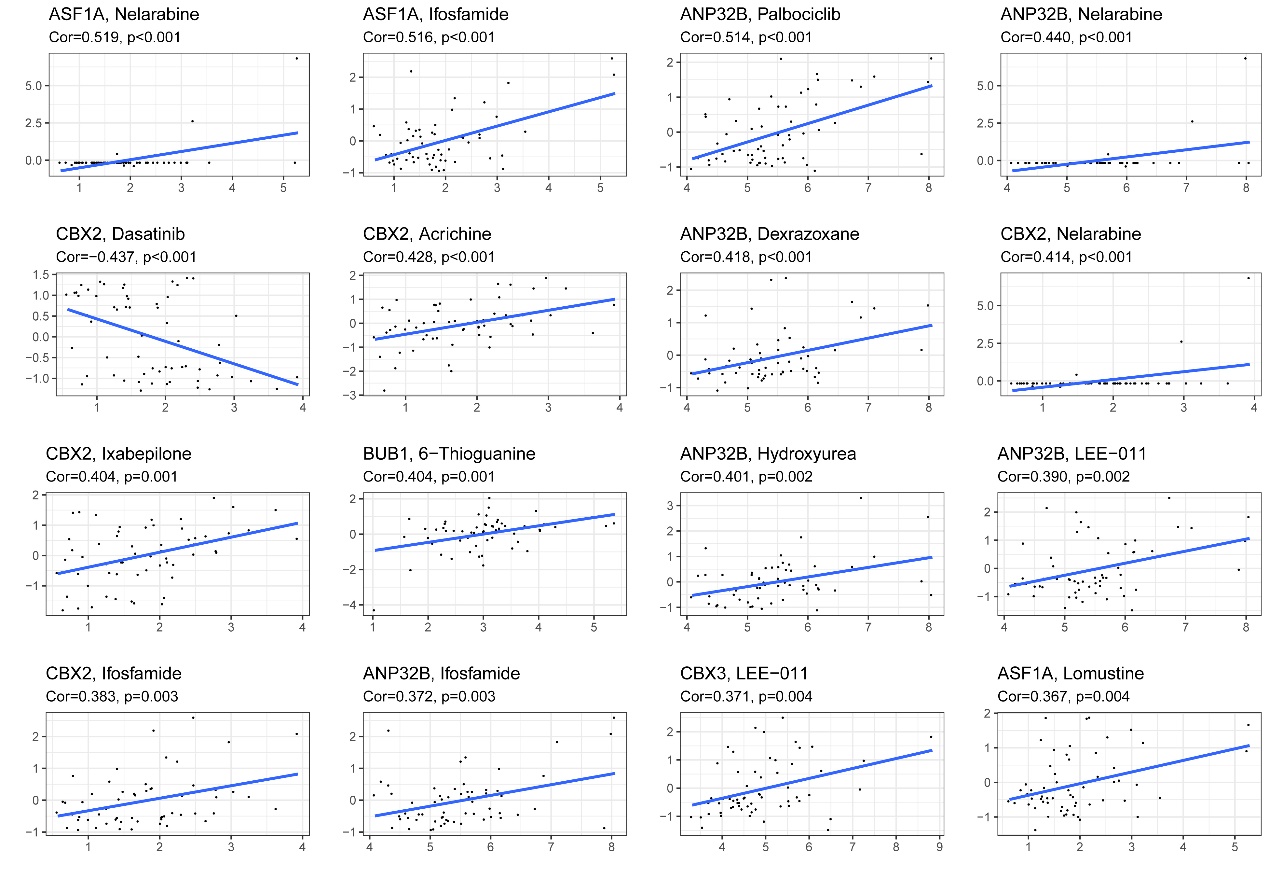


**Figure S4.** Tumor Immune Dysfunction and Exclusion (TIDE) to evaluate the potential clinical efficacy of immunotherapy in different ERGs subgroups. A. TIDE score; B. T cell exclusion score; C. T cell dysfunction score; D. Microsatellite instability (MSI) score; E. ROC analysis the predictive value of our risk model better than 18-gene T-cell-inflamed signature (TIS) and TIDE model.


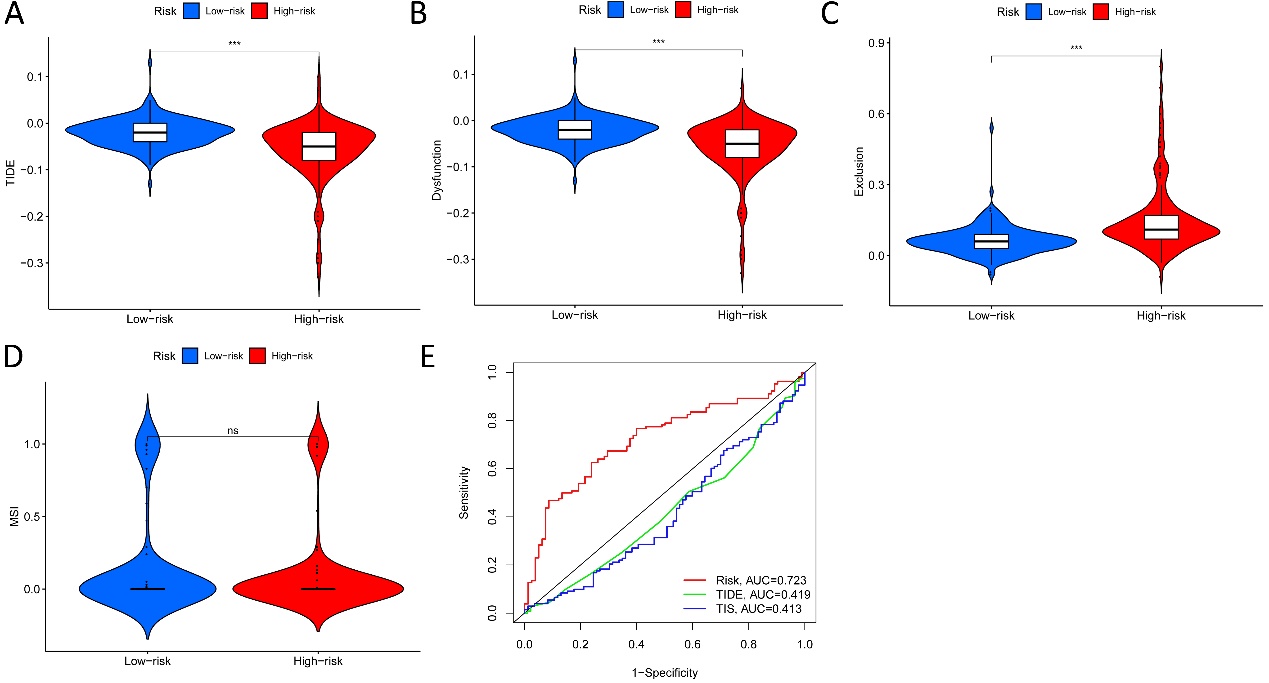


**Figure S5.** The difference in the expression of ICIs and m6A-related mRNA between the high and low risk group. A. ICIs; B. m6A-related mRNA.


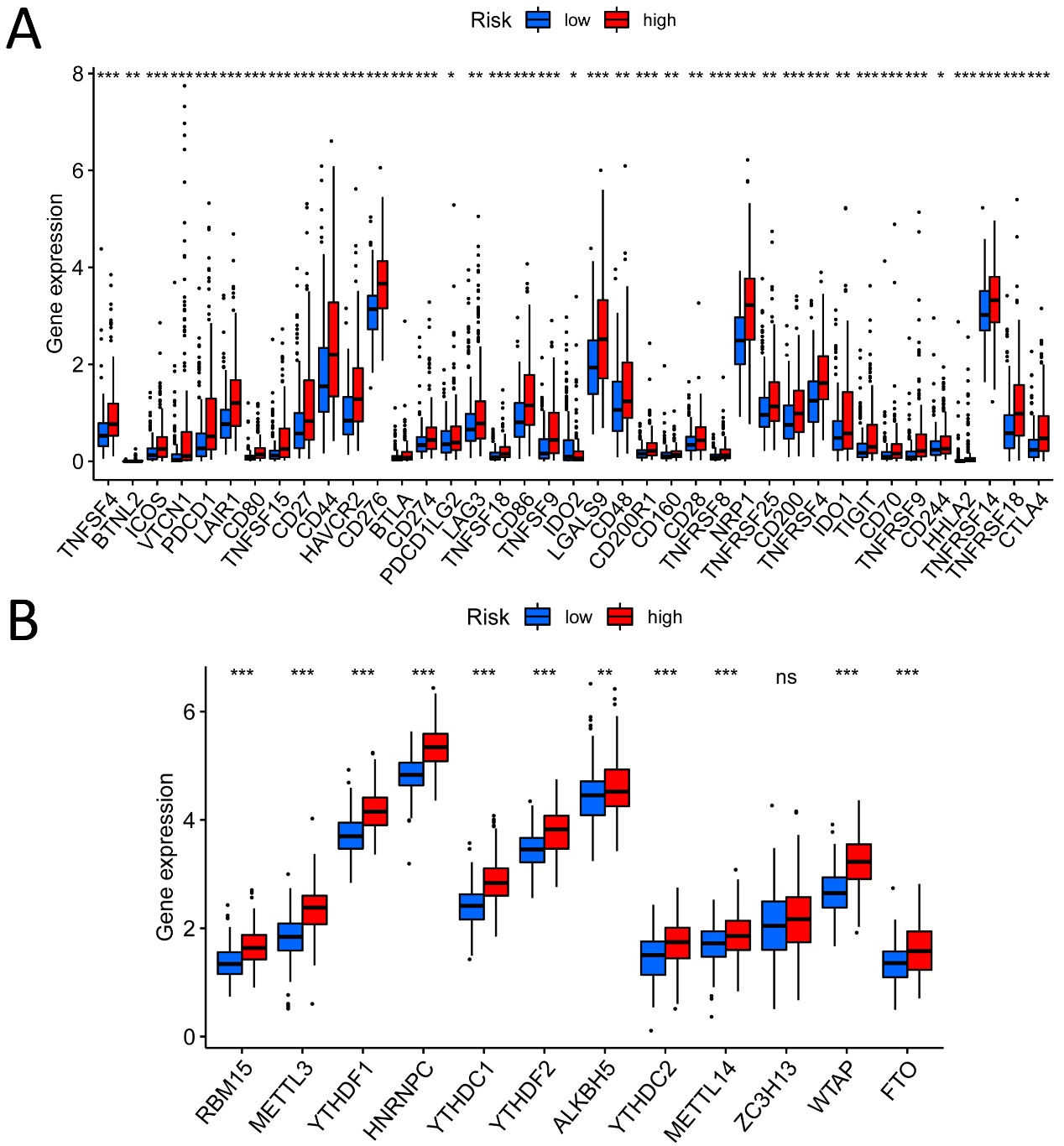


**Figure S6.** The characteristic of the inflammatory response-related genes signature in TCGA. A. Kaplan-Meier curves indicated that high-risk patients had poorer survival than the low-risk group; B. ROC curves to determine whether the expression pattern can be used as an early predictor of HCC; C. The risk survival status chart of patients; D. Univariate Cox analysis; E. Multivariate Cox analysis to identify independent prognosis factors.


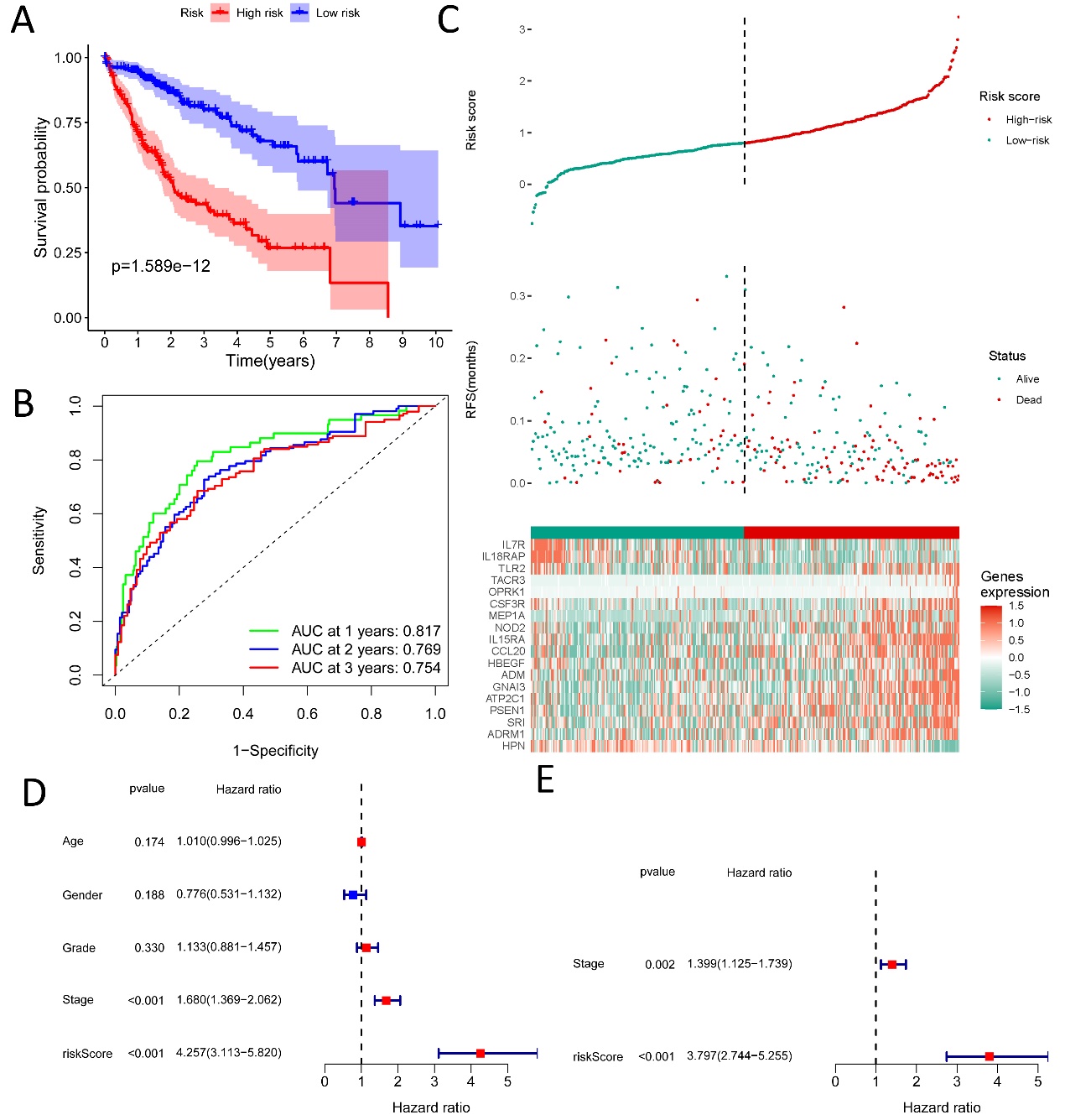


**Figure S7.** The characteristic of the inflammatory response-related genes signature in ICGC. A. Kaplan-Meier curves indicated that high-risk patients had poorer survival than the low-risk group; B. ROC curves to determine whether the expression pattern can be used as an early predictor of HCC; C. Univariate Cox analysis; D. Multivariate Cox analysis to identify independent prognosis factors.


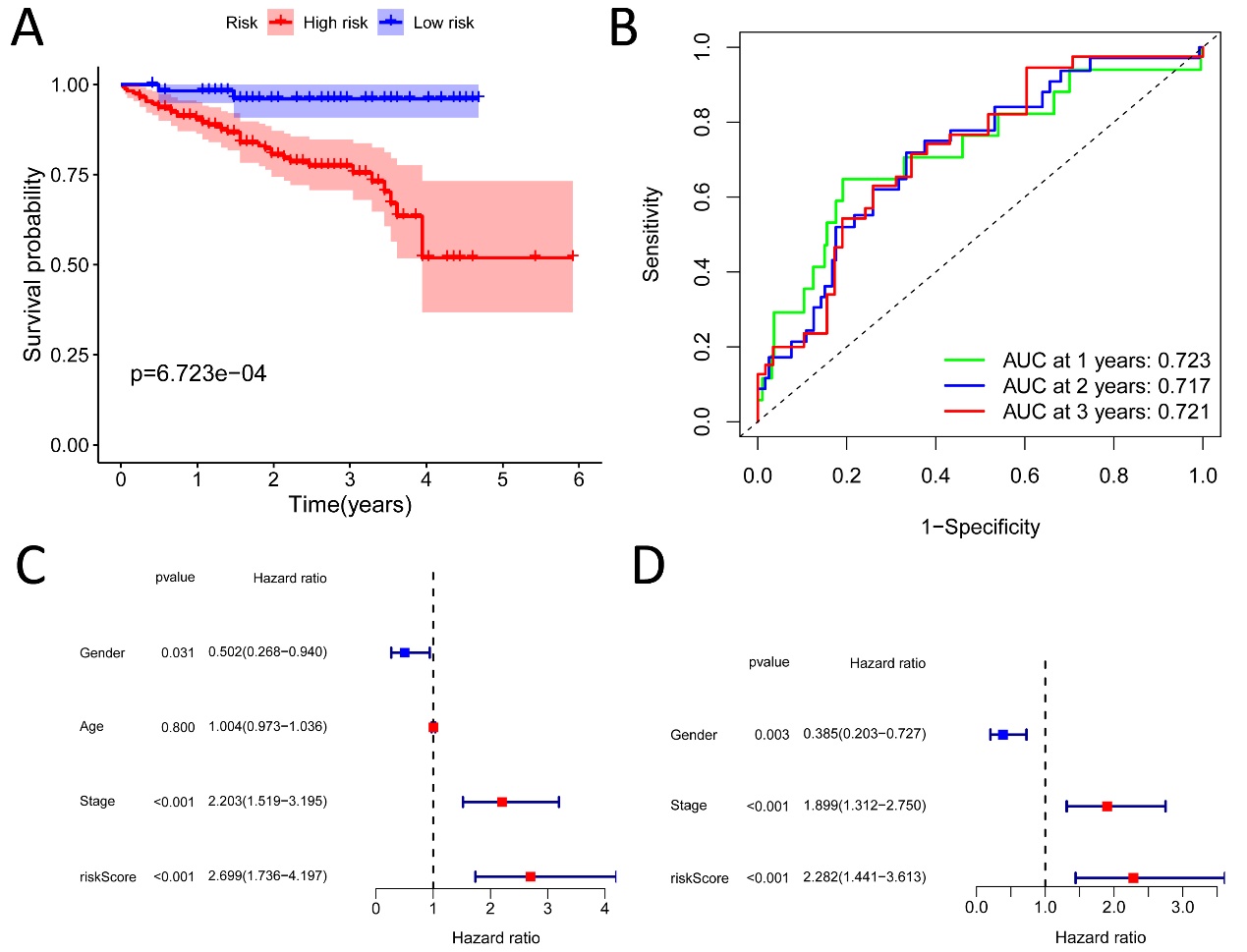

Supplement: Supplementary file 2 [file DataSheet_1.docx]
